# Supplementary material for: CCT8 associates with the MYO10 motor domain and regulates filopodia and breast cancer cell invasion
Source: J Cell Sci. 2026 Sep 29;139(18):jcs265278. doi: 10.1242/jcs.265278 (PMC13676896; doi:10.1242/jcs.265278)
Supplement: Supplementary file 1 [file joces-139-265278-s1.pdf]

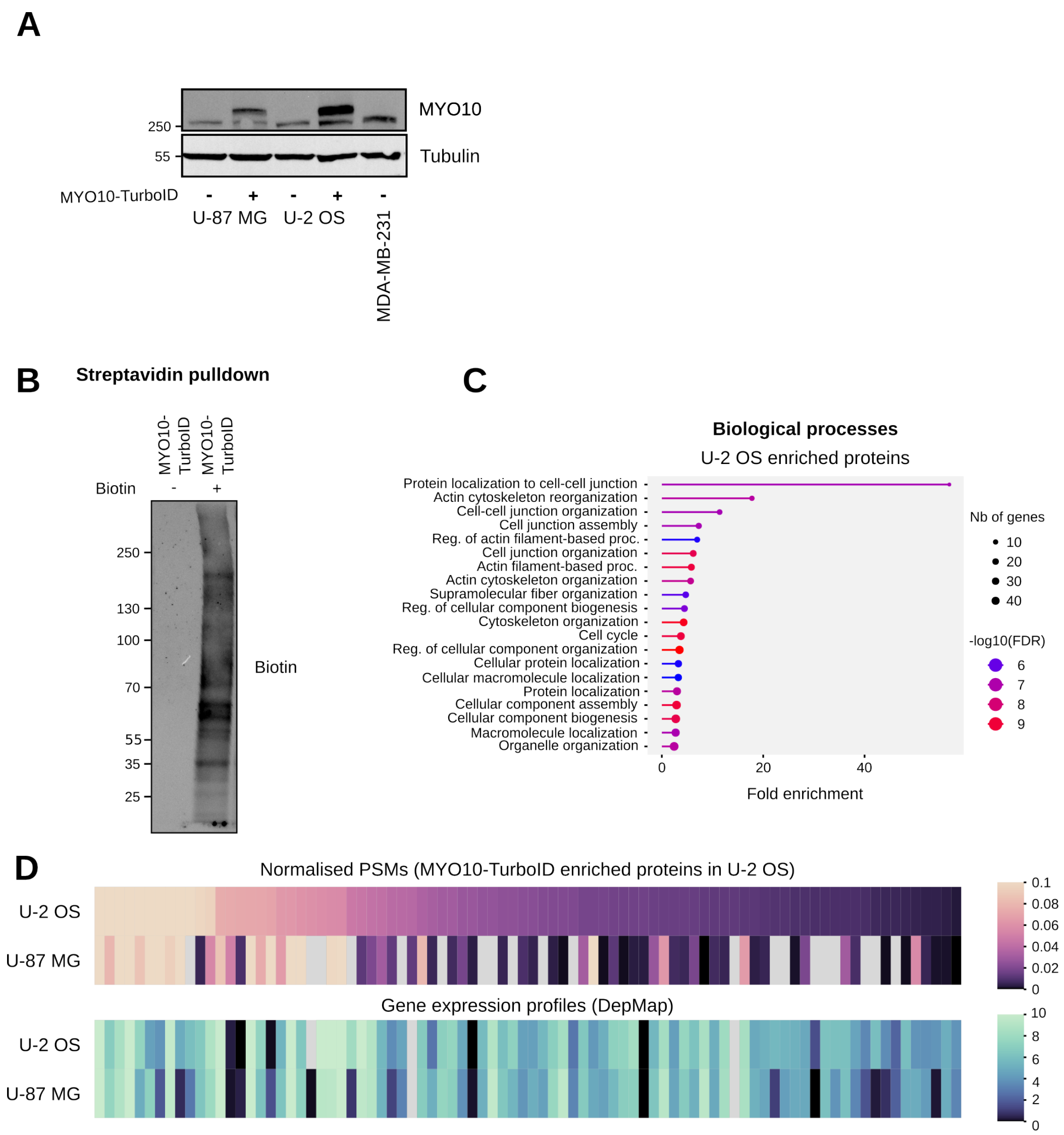

Fig. S1. Identification of MYO10-associated proteins using proximity-dependent biotinylation

(**A**) Representative western blot showing the MYO10 protein levels in cell lines used in this study.
(**B**) U-2 OS cells stably expressing TurboID-MYO10 were cultured on fibronectin-coated dishes for 2 hours, with biotinylation initiated 1 hour before lysis. Biotinylated proteins were affinity-purified using streptavidin-coated beads and analyzed by western blotting.
(**C**) Gene ontology analysis highlighting biological processes significantly enriched among proteins identified in U-2 OS cells.
(**D**) Heatmaps comparing detected MYO10-associated proteins between the two cell lines, specifically highlighting those enriched in the U-2 OS dataset. Expected expression profiles derived from the DepMap database are included for comparison. The numerical data and the raw images used to make this figure have been archived on Zenodo (https://zenodo.org/records/20704995).

Journal of Cell Science • Supplementary information

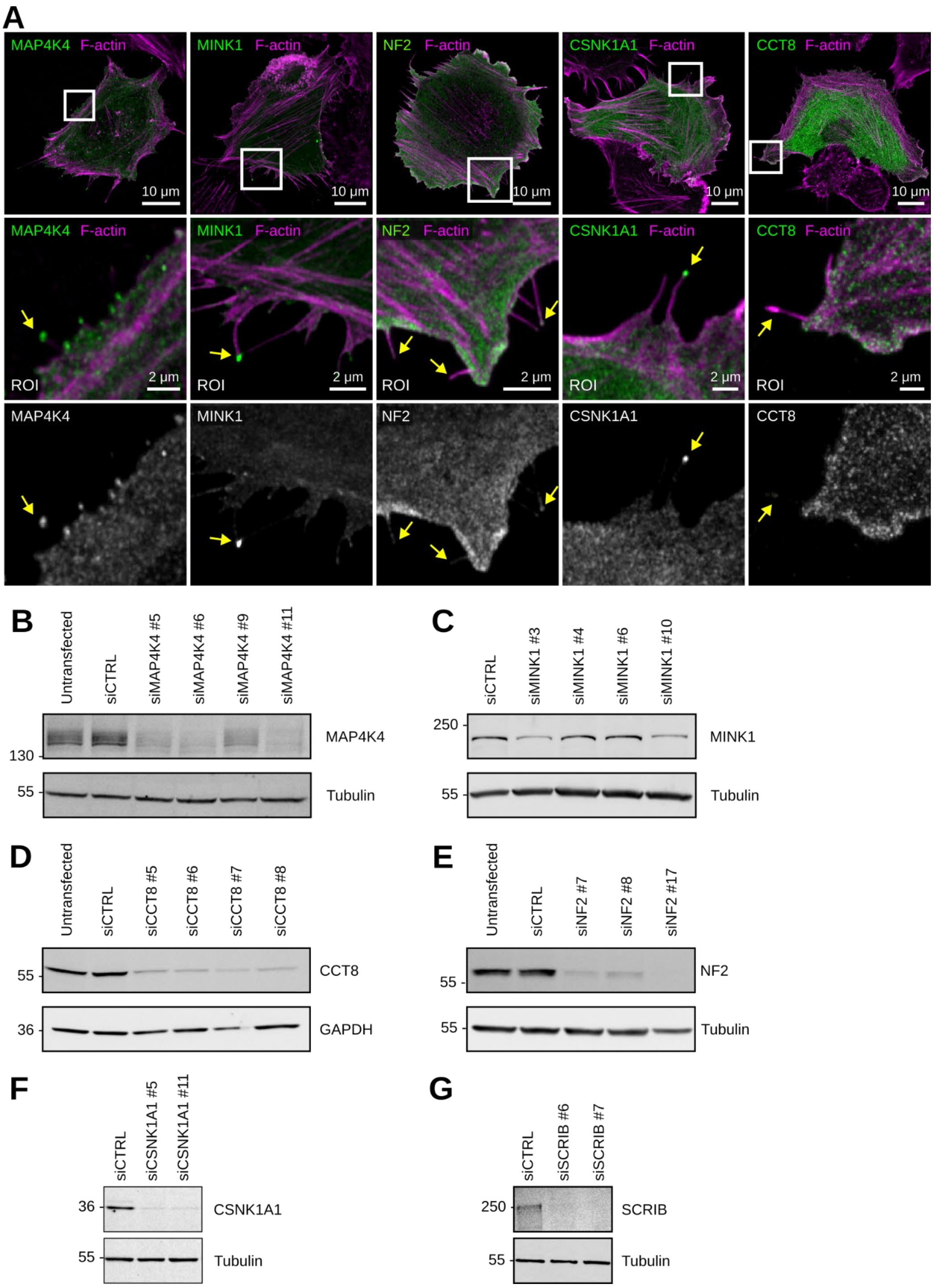

Fig. S2. Candidate protein localization in endogenous filopodia and validation of siRNA-mediated silencing - associated with Fig. 2

(**A**) RAT2 cells expressing the indicated GFP-tagged candidate proteins (MAP4K4, MINK1, CSNK1A1, NF2, or CCT8) were plated on fibronectin for 2 hr, and their endogenous filopodia were stained for F-actin and imaged using Airyscan confocal microscopy. Maximal intensity projections are displayed. Scale bars: Main, 10 µm; ROI, 2 µm.

(**B-G**) U-87 MG cells were treated with multiple siRNAs targeting MAP4K4 (**B**), MINK1 (**C**), CCT8 (**D**), NF2 (**E**), CSNK1A1 (**F**), SCRIB (**G**), or a control siRNA (siCTRL). For each condition, cell lysates were collected, and siRNA efficiency was validated by western blot. Representative western blots are shown. The raw images used to make this figure have been archived on Zenodo (https://zenodo.org/records/20704995).

Journal of Cell Science • Supplementary information

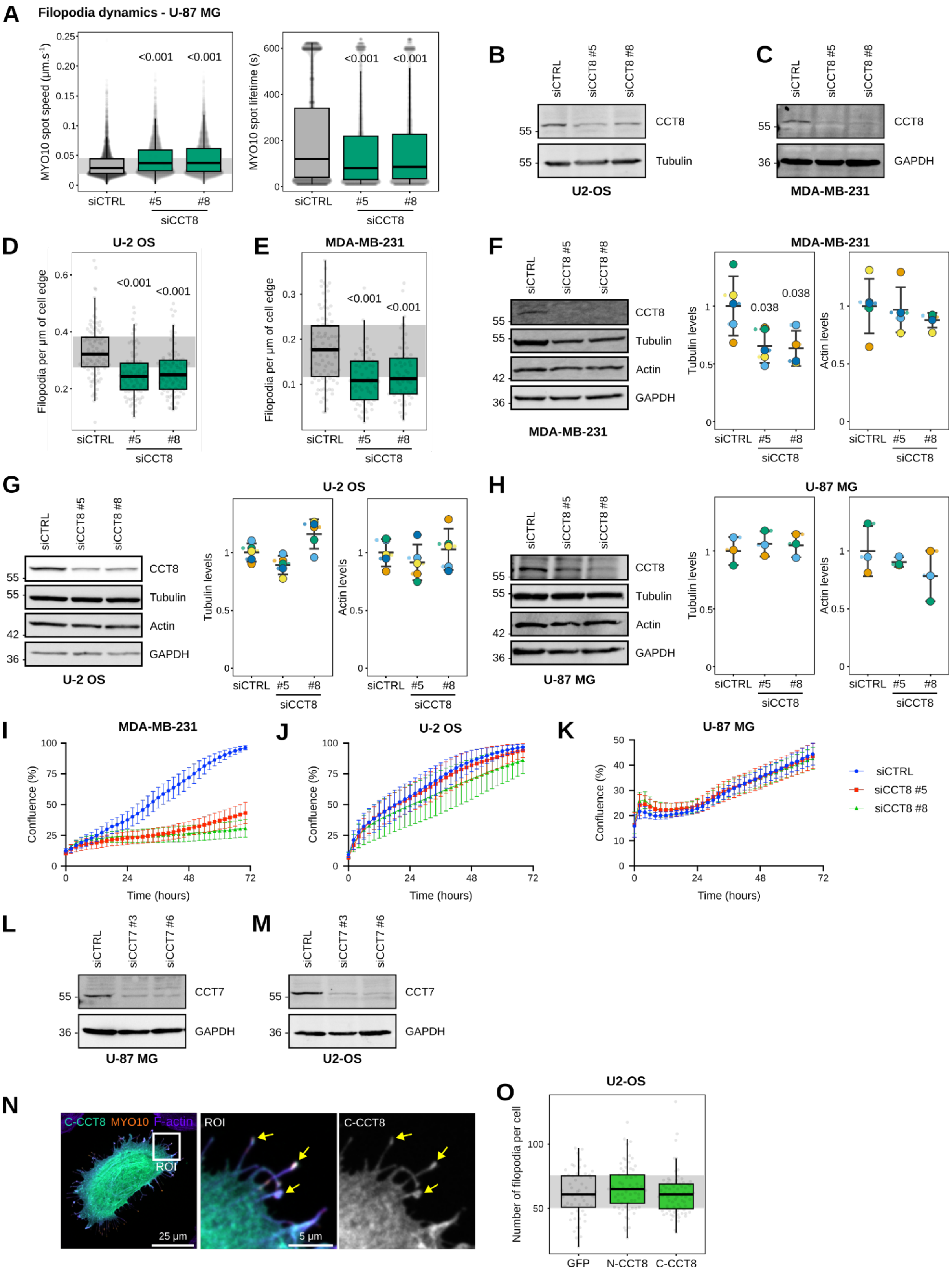

Fig. S3. CCT8 modulates filopodia formation independently of the TRiC complex - associated with Fig. 3

(**A**) CCT8-silenced U-87 MG cells transiently expressing MYO10-GFP were plated on fibronectin and imaged live using an Airyscan confocal microscope, with one image acquired every 5 s for 20 min. MYO10-GFP-positive spots were automatically tracked over time. The reported speeds correspond to the instantaneous velocities of tracked MYO10-positive particles and therefore include movements of MYO10-positive filopodial tips and MYO10-positive spots within filopodia. Track duration was used as a proxy for MYO10 spot lifetime. Average MYO10 spot speed and track duration

Journal of Cell Science • Supplementary information

per cell are shown for three biological replicates, with n > 29 cells per condition. Pooled MYO10 spot speed and lifetime measurements are also shown, from >5000 tracked MYO10-positive particles across three biological replicates.

(**B, C**) U-2 OS (**B**) and MDA-MB-231 (**C**) cells were treated with siRNAs targeting CCT8 or a control siRNA (siCTRL). For each condition, cell lysates were collected, and siRNA efficiency was validated by western blot. Representative western blots are shown.

(**D, E**) Filopodia quantification in U-2 OS (**D**) and MDA-MB-231 (**E**) cells expressing MYO10-GFP, treated with control (siCTRL) or CCT8-targeting siRNAs. Cells were plated on fibronectin-coated coverslips for 2 hours, fixed, and imaged by spinning-disk confocal microscopy. Filopodia per micrometer of cell edge is shown. (**D**) n > 89 cells per condition, three biological replicates. (**E**) n > 61 cells per condition, three biological replicates.

(**F–H**) MDA-MB-231 (**F**), U-2 OS (**G**), and U-87 MG (**H**) cells were treated with CCT8-targeting siRNAs or siCTRL. Cell lysates were collected, and total actin and tubulin levels were assessed by western blotting. Representative western blots and quantification of actin and tubulin levels are shown. Quantifications from independent experiments are presented as SuperPlots, with mean and standard deviation indicated. F and G, n = 5 biological replicates; H, n = 3 biological replicates.

(**I–K**) MDA-MB-231 (**I**), U-2 OS (**J**), and U-87 MG (**K**) cells were treated with CCT8-targeting siRNAs or siCTRL, and proliferation was monitored for 72 h using an Incucyte live-cell analysis system. Confluence over time is shown.

(**L**, **M**) U-87 MG (**L**) and U-2 OS (**M**) cells were treated with siRNAs targeting CCT7 or siCTRL. For each condition, cell lysates were collected, and siRNA efficiency was validated by western blot. Representative western blots are shown. (**N**) Representative confocal images of U-87 MG cells expressing MYO10-mScarlet in combination with CCT8-GFP (C-terminal tag). Cells were plated on fibronectin-coated coverslips for 2 hours before fixation and imaging. Scale bars: Main images, 25 µm; ROIs, 5 µm.

(**O**) Cells expressing mScarlet-MYO10 along with GFP alone, GFP-N-CCT8 (N-terminal tagged), or GFP-C-CCT8 (C-terminal tagged) were treated with siCTRL, plated on fibronectin-coated coverslips for 2 hours, fixed, and imaged using spinning-disk confocal microscopy. Filopodia numbers per cell were quantified. (n > 65 cells per condition, three biological replicates). Results are shown as boxplots, with whiskers extending from the 10th to the 90th percentiles. The boxes illustrate the interquartile range, and a line inside each box indicates the median. Data points outside the whiskers are shown as individual dots. The numerical data and the raw images used to make this figure have been archived on Zenodo (https://zenodo.org/records/20704995).

Journal of Cell Science • Supplementary information

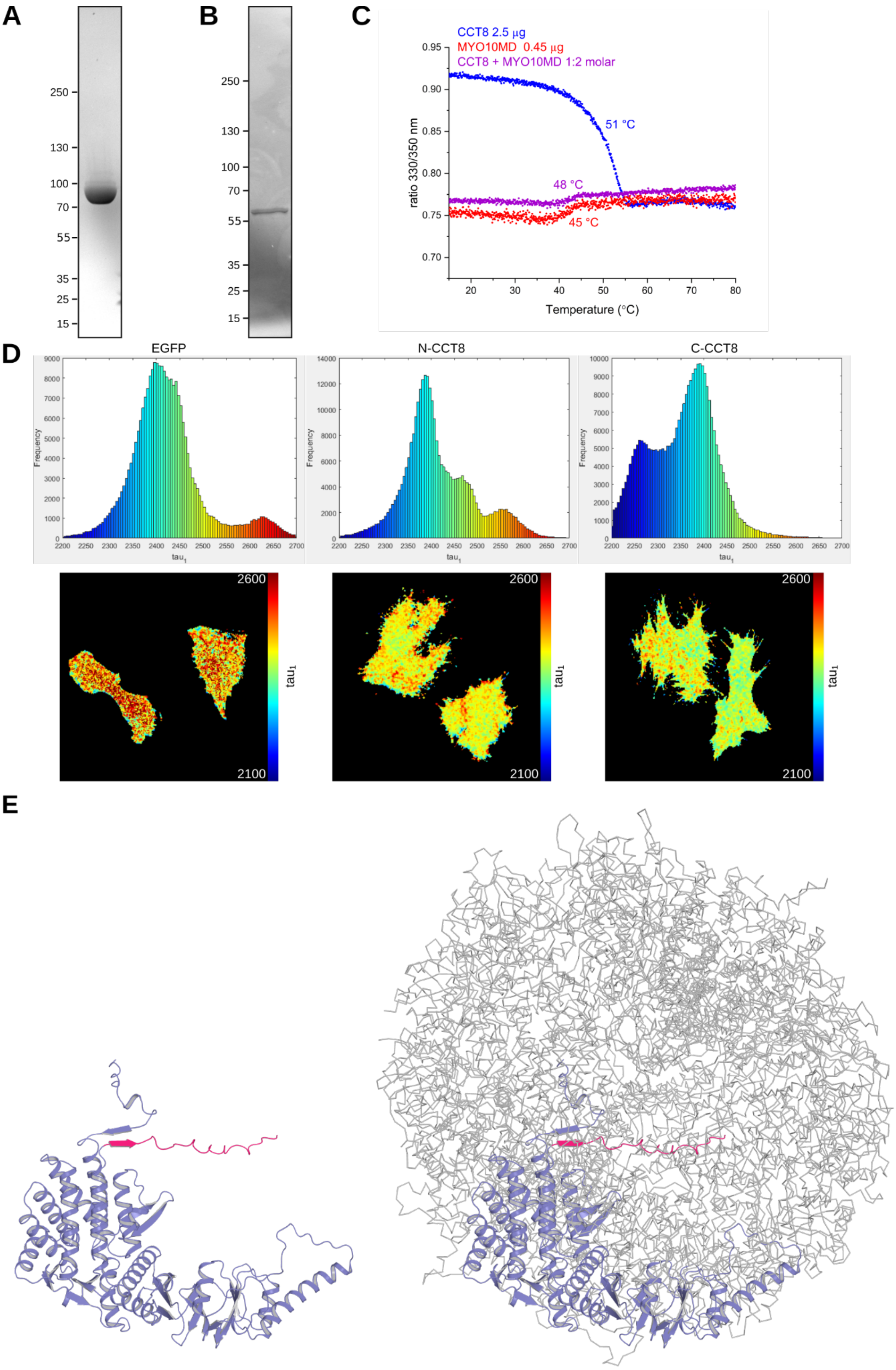

Fig. S4. CCT8 binds to MYO10 - associated with Fig. 4

(**A, B**) Coomassie-stained SDS-PAGE gels showing purified proteins used for in vitro binding assays. (A) MYO10 motor domain purified from insect cells. (B) CCT8 purified from bacteria.

Journal of Cell Science • Supplementary information

(**C**) Thermal unfolding (nanoDSF) profiles of purified CCT8 and MYO10 motor domains (MYO10MD) displaying a Tm of 51 and 45 °C, respectively. The CCT8–MYO10MD complex (1:2 molar ratio) exhibited increased thermostability at 48 °C, compared to MYO10MD alone.
(**D**) GFP lifetime (τ_1) was measured in U-87 MG cells co-expressing MYO10–mScarlet with GFP, GFP–CCT8 (N-terminal tag), or CCT8–GFP (C-terminal tag). Representative images are displayed.
(**E**) The C-terminus of CCT8 (residues 520-548; pink) is exposed (left) or buried (right), depending on whether CCT8 is part of the TRiC complex (6KS6, (Jin et al., 2019)). One copy of CCT8 is shown as a blue cartoon, and the other components of the TRiC complex are depicted as grey ribbons.

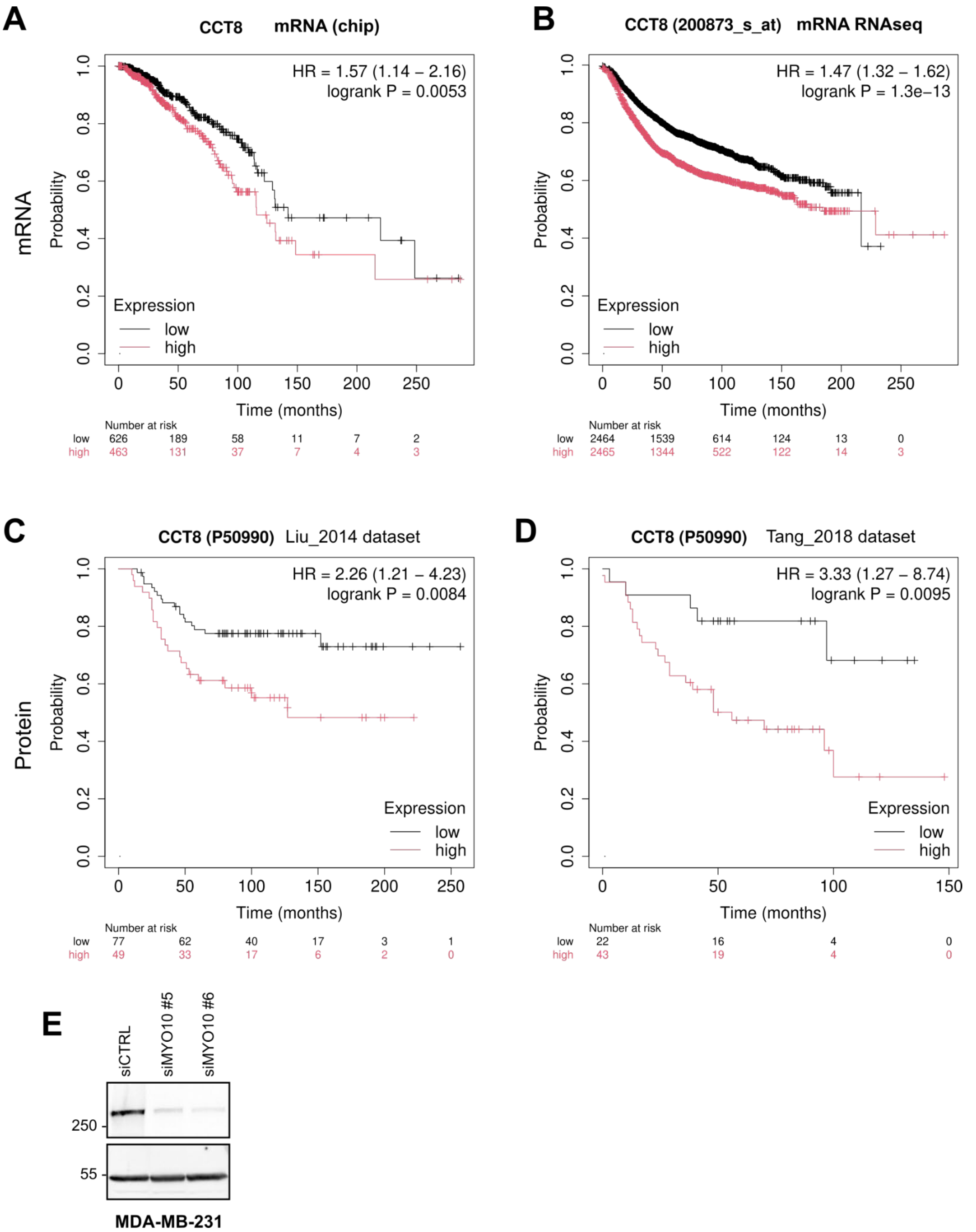

Fig. S5. CCT8 expression and connection to prognosis in breast cancer patients and MYO10 silencing in MDA-MB-231 cells, associated with Fig. 5

(**A**-**D**) Kaplan-Meier survival analyses of breast cancer patients, stratified by high vs low CCT8 mRNA (**A, B**) or protein levels (**C, D**). These analyses were performed using the Kaplan-Meier plotter (Posta and Győrffy, 2025) and four different datasets, as indicated. (**E**) MDA-MB-231 cells were treated with siRNAs targeting MYO10 or with siCTRL. For each condition, cell lysates were collected, and siRNA efficiency was validated by western blot. A representative western blot is shown. The raw images used to make this figure have been archived on Zenodo (https://zenodo.org/records/20704995).

Table S1. Mass spectrometry data.

Available for download at
https://journals.biologists.com/jcs/article-lookup/doi/10.1242/jcs.265278#supplementary-data
